# Prevalence and predictive factors of transient and permanent congenital hypothyroidism in Fars province, Iran

**DOI:** 10.1186/s12887-021-02729-6

**Published:** 2021-06-05

**Authors:** Ashkan Habib, Alireza Shojazadeh, Mohadeseh Molayemat, Asadollah Habib, Marjan Jeddi, Rita Arabsolghar, Mitra Nahas, Nazila Rahimi, Fariba Moradi Ardekani

**Affiliations:** 1 School of Medicine, Shiraz University of Medical Sciences, Shiraz, Iran; 2 Department of Pediatric Endocrinology, School of Medicine, Kazerun Branch, Islamic Azad University, Kazerun, Iran; 3 Endocrinology and Metabolism Research Center, Nemazee Hospital, Shiraz University of Medical Sciences, P. O. Box: 71345-1414, Namazee Square, Shiraz, Iran; 4 Department of Medical Laboratory Science, School of Paramedical Sciences, Shiraz University of Medical Sciences, Shiraz, Iran; 5 Diagnostic Laboratory Sciences and Technology Research Center, School of Paramedical Sciences, Shiraz University of Medical Sciences, Shiraz, Iran; 6 Deputy of Health, Shiraz University of Medical Sciences, Shiraz, Iran; 7 Non Communicable Disease Group, Office of Vice Chancellor for Health Affairs, Shiraz University of Medical Sciences, Shiraz, Iran

**Keywords:** Congenital Hypothyroidism, Iran, Newborn screening, Thyroid, Thyroid-stimulating hormone, Transient vs. permanent congenital hypothyroidism

## Abstract

**Introduction:**

There is no data on the number as well as the prevalence of congenital hypothyroidism (CH) in the Fars province. Hence, we designed this study to analyze the latest data and the possible predictive factors on transient and permanent CH in this province.

**Method:**

This cross sectional study is based on the Fars province screening data from 2013 to 2016. A total of 294,214 newborns were screened with 938 confirmed cases of CH, which were included in this study. After recall and completion of the missing data, follow-up data for 642 CH cases with thyroid stimulating hormone (TSH) concentrations and levothyroxine doses for ten outpatient visits and final transient vs. permanent CH diagnosis were included.

**Results:**

The incidence rate was 1:313.66, and out of the 642 CH cases, 66.04 % had permanent CH, while 33.96 % had transient CH. TSH level trend during the outpatient visits were not statistically different between the two groups (*P* = 0.312). A cutoff point of > 2.25 levothyroxine µg/kg (sensitivity: 76.11 %, specificity: 58.52 %) at the third year and a TSH concentration of > 43.35 mIU/L at the venous sampling (initial TSH) (sensitivity: 31.66 %, specificity: 90.32 %) were the predictive factors for permanent CH.

**Conclusion:**

Fars province has one of the highest incidence rate of CH in Iran. Levothyroxine dose at the 3rd year and the 1st venous TSH sample are the predictive factors for permanent CH in the Iranian population; however, TSH concentrations during follow ups are unreliable predictors.

## Introduction

It is well known that thyroid hormones play a vital role in the maturation of human brain via neural cell migration, differentiation and signaling [1]. The most crucial effect of thyroid hormones is on the central nervous system, particularly during the first two years of life [2]. The clinical manifestations of congenital hypothyroidism (CH) at birth are relatively mild and the clinical suspicion of the disease appears in less than 1 % of the affected newborns. Thus, a missed diagnosis of CH in neonates can ultimately result in irreversible complications, such as deafness or intellectual disabilities [3–5]. Therefore, it is necessary to design a screening program to detect CH during the early phases of the disease by measuring T4 (thyroxine) or TSH (thyroid stimulating hormone) in neonates [6]. A prompt thyroid hormone replacement in newborns with Levothyroxin will lead to normal neurologic growth [7]. The incidence of CH has more than doubled in the recent years, which is due to the introduction of more comprehensive diagnostic criteria, shifting demographic and increased survival rate of preterm newborns [8]. For instance, a cohort study reported the prevalence rates of 1 per 4,094 births in the USA in 1987, which increased to 1 per 2,372 births in 2002 [9]. Reports from Iran indicate 1 per 914 live births in Tehran to 1 per 357 live births in Isfahan [10, 11]. These reports reveal much higher rate than the 1 in 4000 in North America, Europe and Australia [12, 13].

According to temporary vs. lifelong treatment of the disease, congenital hypothyroidism is divided in two categories of transient and permanent. The transient form is due to exposure of the placental to antithyroid medications or antithyroid receptor antibodies, excess or deficiency in iodine level, preterm neonates with very low birth weight, thyroid iodine organification immaturity, or DUOX2 mutations. However, permanent CH is mostly caused by thyroid agenesis or dyshormonogenesis [14, 15]. In Iran, transient CH rate amongst the confirmed cases varies from 56.46 % in Mazandaran to 36 % in Hamadan [16–19], which is much higher than the 10–15 % reported by the other countries [20–22].

To the best of our knowledge, there is no update on the prevalence of CH, figures or the distribution of transient vs. permanent CH in the Fars province. As a result, we designed this study to provide a more accurate analysis of the latest data and the possible predictive factors for transient and permanent CH in Iran.

## Method

This cross sectional study is based on Fars provincial screening data from March 2013 till March 2017, which is situated in south west of Iran. Data were obtained from the office of non-communicable diseases at Shiraz University of Medical Sciences (SUMS). The study was approved by the SUMS medical research ethics committee. (Code:IR.SUMS.MED.REC.1398.280)

The Fars province screening program was established in 2004. The program is based on the ‘National Congenital Hypothyroidism Screening Program Guidelines’ designed by the Iranian Ministry of Health. To ensure 100 % newborn coverage, all newborns in the Fars province are introduced to the program. An in-depth look revealed more than 99 % neonatal coverage in the study period [23]. Within 3 to 5 days of birth, a heel prick blood sample is taken on 903 Whitman filter papers. Samples are sent by express mail to the Shiraz provincial screening center and TSH levels are tested, using the DiaZist (Tehran, Iran) ELISA kits. Patients with TSH levels between 5 and 10 IU/ml in the first round of screening along with premature neonates, low birth weight (< 2500 gr) and very low birth weights (< 1500 gr), newborns with birth weights above 4000 gr, twins and multiple births, hospital admitted neonates, familial history of thyroid disorders, drug usage, such as corticosteroid, dopamine or patients with history of blood transfusion or exchange transfusions are recalled and reevaluated, using the heel prick sampling, even with normal TSH in the first rounds within 8 to 14 days of birth. Patients with TSH levels ≥ 10 IU/ml in the first round and ≥ 5 IU/ml in the second round of screening are sent to an approved pediatrician for a thorough evaluation as well as venous TSH test for a confirmation of their CH. Neonates with total T4 < 6.5 µg/dL and TSH > 10 mIU/L are diagnosed as CH with the start of treatment thereafter. The confirmed CH cases are reported by the pediatrician to the office of non-communicable diseases in Shiraz University of Medical Sciences. These patients are closely monitored by a pediatric endocrinologist 2 to 4 weeks after the commencement of therapy, then every two months in the first 6 months and every three months thereafter till 3 years of. If TSH levels become normal during this period, patients undergo a discontinuation of levothyroxine therapy for a month, and then TSH and T4 levels are retested. If TSH level does not rise, they are labeled as transient CH; otherwise they are considered as permanent and therapy will be continued for the rest of their life.

According to the screening data, which are demonstrated in Table 1, during the study time period, 294,214 newborn were screened with 938 confirmed cased of CH. Participant’s sex, height and birth weight, date of birth, parental consanguinity, familial thyroid disorders, TSH concentrations at first and second screenings, the confirmatory venous sampling along with the age at which the samples were taken, the initial dose of treatment as well as TSH and LT4 dose per kilogram for 10 follow-ups were recorded for this study.

**Table 1:** Description of the Fars provincial neonatal program for congenital hypothyroid screening from 2013–2016

Year	Total number of newborns screened		TSH (5-9.99 mIU/L)		TSH (10-19.99 mIU/L)		TSH (≥ 20 mIU/L)		Confirmed Congenital Hypothyroidism	Confirmed Transient Congenital Hypothyroidism (n, %)	Confirmed Permanent Congenital Hypothyroidism (n, %)
	First round	Second round	First round	Second round	First round	Second round	First round	Second round			
**2013**	72,192	12,235	1133	145	83	36	21	21	182	36 (27.9 %)	93 (72.1 %)
**2014**	74,383	14,601	1109	186	86	32	37	8	227	68 (41 %)	98 (59 %)
**2015**	74,643	14,512	1396	281	43	27	33	4	258	56 (30.3 %)	129 (69.7 %)
**2016**	72,996	19,582	1685	390	41	26	21	10	271	58 (35.8 %)	104 (64.2 %)

**Abbreviations**: *TSH* thyroid stimulating hormone

For some patients, results from the 3 years of follow-up were missing, and the most likely causes were that some patients had either expired, migrated out of the province, abruptly stopped following their physicians recommendations or had changed their physician without prior notice. Hence, to extent that was possible, we recalled the patients with incomplete follow-up data for better conclusive results. Finally, 642 CH patients had final transient vs. permanent diagnosis after 3 years of follow up, which were included in this study.

**Fig. 1:**
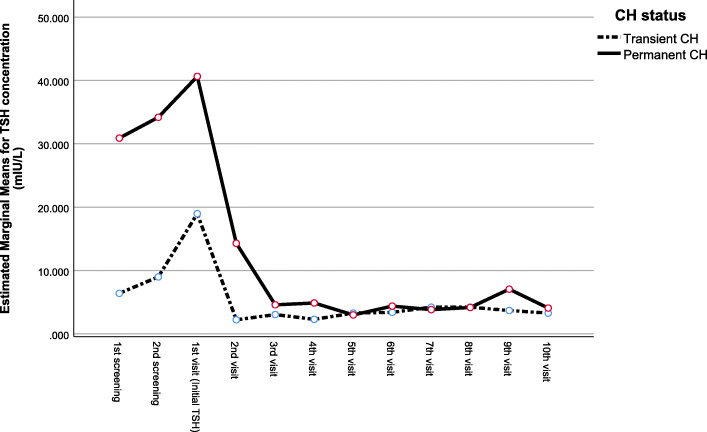
Repeated measures ANOVA of twelve consecutive TSH measurements from 42 permanent and 8 transient CH cases.

**Fig. 2:**
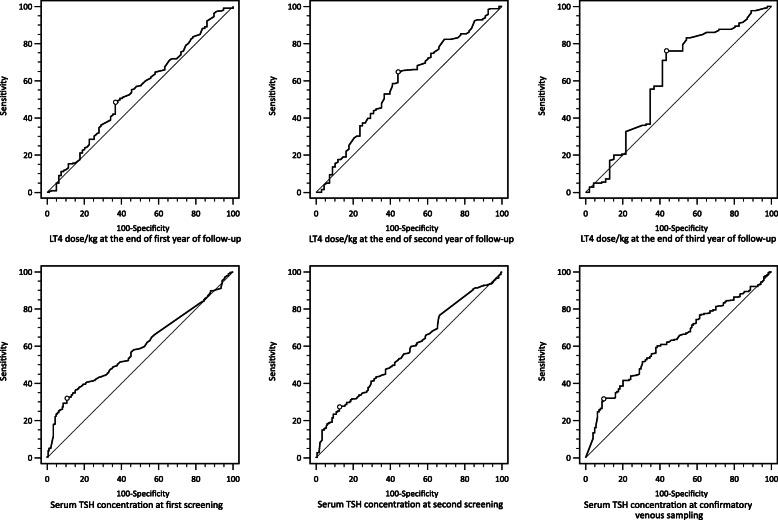
Receiver operating characteristics (ROC) for predicting permanent CH based on TSH levels (mIU/L) and LT4 dose (μg/kg). Circles on curve lines represent optimal cutoffs based on Youden’s index.

### Statistical analysis

Comparisons were made, using Student t-test for quantitative variables and Chi-square for qualitative variables in Table 2. Statistical significance for difference in TSH concentration trends after outpatients visits were performed, using General Linear Model Repeated Measures ANOVA (Fig. 1). Analysis for prediction of permanent CH was done, using TSH concentration during screening. Confirmatory venous samplings and levothyroxine dosage at the end of each year of follow up were performed, using receiver operating characteristic curve (ROC) curve (Fig. 2; Table 3). The optimum cutoff point for each predictive factor were calculated based on Youden’s index. A *P* value of < 0.05 was considered to be statistically significant in all comparisons with a confidence interval of 95 %. Statistical analysis for all tables and Fig. 1 were performed, using SPSS software version 25.0 (SPSS, Chicago, IL, USA) and MedCalc software version 14.8.1 (MedCalc, Ostend, Belgium) for ROC curves in Fig. 2 and Youden’s index.

**Table 2:** Characteristics of Transient and Permanent Congenital Hypothyroidism

Variables		Permanent CH NO (%) mean ± SD	Transient CH NO (%) mean ± SD	*p*-value
Sex	Male	222 (52.4 %)	116 (53.2 %)	0.838
	Female	202 (47.6 %)	102 (46.8 %)	
Birth Weight (g)^a^		2912.8 ± 629.1 (*N* = 418)	2931.1 ± 666.7 (*N* = 216)	0.733
Height^a^		48.8 ± 3.9 (*N* = 407)	48.5 ± 4.0 (*N* = 198)	0.475
Parental consanguinity^a^	Absent	182 (44.7 %)	118 (60.8 %)	< 0.001*
	Present	225 (55.3 %)	76 (39.2 %)	
Age at start of treatment (day)		25.19 ± 25.14	26.39 ± 22.34	0.583
Initial LT4 dose (µg)		41.77 ± 22.59	39.36 ± 22.07	0.232
TSH level at first measurement	Age at measurement (days)	5.58 ± 5.72	5.14 ± 3.88	0.346
	TSH concentration level (mIU/L) mean ± SD (Median, IQR)	28.33 ± 47.62 (6.70, 5.00–19.60)	11.43 ± 25.27 (5.65, 5.00–8.73)	< 0.001*
TSH level at second measurement	Age at measurement (days)	14.25 ± 9.98	14.65 ± 8.58	0.664
	TSH concentration level (mIU/L) mean ± SD (Median, IQR)	25.00 ± 39.55 (8.20, 5.20–20.42)	13.8 ± 24.56 (7.10, 5.00–12.63)	< 0.001*
Season^a^	Spring	87 (21.4 %)	48 (23.4 %)	0.849
	Summer	98 (24.1 %)	46 (22.4 %)	
	Autumn	124 (30.5 %)	58 (28.3 %)	
	Winter	98 (24.1 %)	53 (25.9 %)	
Method of delivery^a^	Normal vaginal delivery	194 (46.7 %)	113 (57.4 %)	0.014*
	Cesarean section	221 (53.3 %)	84 (42.6 %)	
Family history of CH^a^	Absent	306 (74.6 %)	145 (74.4 %)	0.942
	Present	104 (25.4 %)	50 (25.6 %)	
Serum TSH concentrations by each visit (mIU/L)^a^ mean ± SD (Median, IQR)	First visit (confirmatory venous TSH, Initial TSH)	34.51 ± 31.51 (20.00, 10.50–55.30) *N* = 259	21.12 ± 21.98 (12.95, 8.33–23.35) *N* = 124	< 0.001
	Second visit	13.92 ± 27.22 (4.48, 1.04–12.00) *N* = 201	8.86 ± 15.99 (2.90, 1.47–8.27) *N* = 85	0.052
	Third visit	5.68 ± 12.19 (2.38, 0.92–5.41) *N* = 198	4.51 ± 9.77 (1.77, 0.49–3.40) *N* = 71	0.467
	Fourth visit	4.89 ± 9.18 (2.59, 1.00–5.00) *N* = 191	3.43 ± 4.19 (2.10, 1.20–4.48) *N* = 67	0.212
	Fifth visit	4.23 ± 7.76 (2.00, 0.80–5.35) *N* = 181	2.79 ± 1.95 (2.44, 1.36–3.65) *N* = 64	0.023*
	Sixth visit	4.48 ± 9.04 (2.50, 1.28–4.43) *N* = 174	3.32 ± 2.44 (2.94, 1.68–4.29) *N* = 64	0.122
	Seventh visit	4.80 ± 6.96 (2.90, 1.50–5.43) *N* = 170	3.08 ± 2.40 (3.06, 1.28–3.84) *N* = 56	0.006*
	Eight visit	5.09 ± 9.33 (2.57, 1.36–4.70) *N* = 179	3.39 ± 3.47 (2.90, 1.51–4.27) *N* = 49	0.049*
	Ninth visit	6.17 ± 14.68 (2.83, 1.24–5.28) *N* = 144	3.03 ± 1.41 (3.39, 1.96–3.83) *N* = 44	0.013*
	Tenth visit	8.09 ± 38.14 (2.60, 1.20–5.10) *N* = 155	3.25 ± 1.42 (3.23, 2.01–4.47) *N* = 40	0.424
LT4 dose by end of each year (µg/kg)^a^	First year	4.69 ± 2.38 (*N* = 221)	4.48 ± 2.73 (*N* = 79)	0.521
	Second year	4.36 ± 2.24 (*N* = 198)	3.82 ± 2.43 (*N* = 68)	0.097
	Third year	3.99 ± 1.99 (*N* = 180)	3.37 ± 2.32 (*N* = 46)	0.072

*p < 0.05

^a^Total number do not add up to 642 due to missing data.

Abbreviations: *CH* congenital hypothyroidism; *SD* standard deviation; *LT4* levothyroxine; *TSH* thyroid stimulating hormone; *IQR* inter quartile range

**Table 3:** Possible predictive factors for distinguishing permanent congenital hypothyroidism based on receiver operating characteristics (ROC)

Variable	AUC	P	Optimal cutoff	Sensitivity	Specificity
**LT4 dose by weight at 1st year**	0.545	0.241	> 3.91 µg/kg	48.42 %	63.29 %
**LT4 dose by weight at 2nd year**	0.587	0.033*	> 2.94 µg/kg	64.65 %	55.88 %
**LT4 dose by weight at 3rd year**	0.616	0.026*	> 2.25 µg/kg	76.11 %	58.52 %
**TSH at 1st screening**	0.593	< 0.001*	> 13.2 mIU/L	31.92 %	89.32 %
**TSH at 2nd screening**	0.578	0.002*	> 18.5 mIU/L	27.22 %	87.36 %
**TSH at confirmatory venous sampling (Initial TSH)**	0.625	< 0.001*	> 43.35 mIU/L	31.66 %	90.32 %

*p < 0.05

**Abbreviations**: *AUC* area under curve; *LT4* levothyroxine; *TSH* thyroid stimulating hormone

It should be noted that since we have used the Persian calendar, throughout this article we will be referring to each year from March of that year to the next. For example, when referring to 2015, the time period is from March 2015 to March 2016.

## Results

Table 1 shows an overall view of the screening program in each year. From March 2013 till March 2017, 294,214 neonates were screened in the first round with 60,930 in the second round with a 20.71 % re-test rate. A total of 938 subjects had confirmed congenital hypothyroidism. The incidence rate for the overall time period is 1:313.66. An overview of the data reveals that 424 (66.04 %) children in this study had confirmed permanent CH, while 218 (33.96 %) had transient CH. Although the incidence of confirmed CH patients has statistically increased each year (*p* < 0.001), and 2016 having the highest number of confirmed CH, the proportion of permanent CH in each year does not follow a specific trend, and it has a relatively minor fluctuation range.

Table 2 shows the characteristics of patients with permanent vs. transient CH. Patients with permanent CH had significantly higher proportion of parental consanguinity than the transient patients. Adjusted for sex, permanent CH patients were 1.917 times more likely to have parental consanguinity than the transient patients (OR = 1.917, CI 95 %:1.353–2.716, *p* < 0.001). Cesarean section delivery was also more prevalent amongst the permanent CH vs. transient CH. Adjusted for sex, permanent CH patients were 1.535 times more likely to have been delivered via C-Section in comparison to transient CH patients (OR = 1.535, CI 95 %:1.089–2.163, *p* = 0.014). TSH concentrations were significantly higher in the permanent CH patients during the first and second round of screenings. TSH serum levels are generally higher in permanent CH group during all outpatient visits, but only in the course of the 5th and 7th to 9th visit they reach statistical significance. The difference in the rest of outpatient visits are statistically insignificant. In total, 54.9 % of the permanent CH patients had experienced at least one uncontrolled TSH level (TSH ≥ 5) at some point from the 3rd visit onwards vs. 36 % amongst the transient CH patients (*P* < 0.001).

Figure 1 shows the overall view of TSH serum mean level concentration after the first and second screenings and following the outpatient visits for 50 subjects (8 transient CH, 42 permanent CH) with complete information of ten consecutive and orderly follow-ups. Repeated measures ANOVA with a Greenhouse-Geisser correction shows that despite the significant reduction in TSH concentration during the twelve consecutive measurements (F(2.092, 100.436) = 3.606, *P* = 0.029), this trend in TSH serum concentration in the transient vs. permanent CH is statistically indifferent (F(2.092, 100.436) = 1.182, *P* = 0.312) Post-hoc test, using Bonferroni correction revealed a steady, but statistically insignificant rise in TSH concentration from the first screening until the confirmatory venous TSH level during the first visit (26.97 ± 57.92 mIU/L vs. 37.16 ± 32.76 mIU/L, respectively. *p* = 1.000) followed by a statistically significant drop until the third visit (37.16 ± 32.76 mIU/L vs. 4.34 ± 5.66 mIU/L, respectively. *p* = 0.007) for both categories of CH. However, Serum TSH concentration in both categories remained relatively steady and within similar range throughout the next seven follow-ups till the tenth visit (4.34 ± 5.66 mIU/L vs. 3.95 ± 5.62 mIU/L, respectively. *p* = 1.000).

According to the results, shown in Fig. 2; Table 3, overall, levothyroxine dose by weight as a predictor for permanent CH only reaches statistical significance at the second and third year of follow up. Meanwhile, all three serum TSH concentrations had statistically significant area under curves (AUC) and were indicators of permanent CH vs. transient.

## Discussion

In this study, the incidence of CH in Fars province from 2013 to 2016 was 1:313.66. In Iran, the incidence rate was 1:914 live births in Tehran [10], 1:357 in Isfahan[11], 1:1608 in Yazd [17] 1:1250 in Hamadan[19] 1:446 in Ahvaz [18] 1:453 in Mazandaran [16], and 1:307 in Markazi [24]. Our result is in line with Isfahan and Markazi provinces. The prevalence of CH for the rest of the world is 1:2118 in Alabama (a USA state) [25], 1:1684 in Oregon (a USA state) [26], 1:469 in Turkey [27], and 1:834 in Saudi Arabia [28]. The obtained results in Iran are far higher than the incidence rates in other parts of the world, which can be due to the low cut-offs for CH suspicion in the screening tests, the Iranian gene pool, ethnicity, autoimmune factors, parental consanguinity, and iodine deficiency [14]. Due to cultural differences, parental consanguinity is more accepted in Iran. In addition, several studies showed that dyshormonogenesis, which is mainly caused by autosomal recessive inheritance, has higher prevalence rate amongst the CH patients in Iran [11, 18, 29]. Furthermore, the high neonatal TSH re-test rates (20.71 %), the moderate iodine deficiency in Iranian pregnant women [30] and the low TSH cut-off values in both screening tests all contribute to the higher rates of transient CH in Iran.

A 2007 study on CH in Fars province revealed an incidence rate of 1:1465 [29]. Aside from the higher incidence rate in our study, we found a statistically significant increase in the prevalence of CH, even within the period, from 2013 to 2016. Shaghaghian et al. argue that the increase in the rate of CH prevalence in Fars can be attributed to better coordination between responsible organizations for the screening program, increased access to screening centers, increased doctor to patient ratio in the province, better training programs for pregnant women and the implementation of screening regulations for hospitalized neonates [23].

Permanent vs. transient CH rates differ widely in the world and even within Iran. In Yazd, 45.5 % of the confirmed CH cases were permanent [17], in Hamadan 64 % [19], in Ahvaz 46 % [18], in Isfahan 59.8 % [11] and in Markazi 51.9 % [24]; while the rest of CH cases were transient. The last study in Fars province (2007) reported 53.6 % permanent CH cases [29]. Worldwide, 66.44 % of CH cases in the state of Alabama (USA) [25], 70.6 % in the state of Oregon (USA)[26], 75 % in the state of Michigan (USA)[22], 62 % in France [31], and finally, 46.6 % of CH cases in China were permanent; while the rest were transient [32]. In our study, 66.04 % had confirmed permanent CH, while only 33.96 % had transient CH. Our results are more in line with studies from Hamadan and Isfahan in Iran, and Alabama (USA) and France. The difference in permanent CH rate worldwide can be attributed to iodine status and most importantly, differences in CH screening methods. For example, states in the USA use two round primary T4-reflex TSH method instead of the primary TSH method, which is used in Iran. Although in recent years, most state programs in the US have switched to the primary TSH method. Differences between provinces inside Iran can be mainly attributed to iodine levels. Despite successful universal salt iodization program since the 1990 s and improvement in Iranian iodine status, there still remains a moderate iodine deficiency amongst the Iranian pregnant women [30]. Transient CH rate in Fars province has also decreased from 46.4 % to 2007 [29] to 33.96 % in our study, which can be explained by the improvement in urine iodine status in the Fars population [33].

Effects of parental consanguinity and familial history of thyroid disorders on transient and permanent CH is controversial. While studies by Rabbiosi et al., Dorreh et al., and Saba et al., found first-degree familial thyroid disorders more prevalent in permanent CH patients [24, 34, 35], Zhou et al., found them to be more prevalent in transient CH [32]. In our study, we did not find any statistical differences in the prevalence of familial thyroid disorders between the two groups, although it should be noted that both were higher than the general population. Familial thyroid disorders can account for transient CH based on maternal autoimmune diseases and trans-placental passage of autoimmune antibodies, while goiter and nodular disorders in familial history can account for permanent CH [34]. In our study, similar to Saba et al., parental consanguinity was more frequent in permanent CH[35], while in studies by Dorreh et al. ., and Razavi et al., there were no statistical difference between the two groups [19, 24]. We also found caesarian section delivery to be more prevalent amongst permanent CH. The same result was obtained by Dorreh et al., in Markazi province [24] However, Rabbiosi et al., found no significant difference with respect to the method of delivery between the two groups [34].

In our study, the trend in reduction of TSH concentration in the span of ten outpatient visits was not statistically different between the transient vs. permanent CH; nevertheless, we found TSH serum concentration level to be generally higher throughout all visits in permanent CH cases. A closer look at TSH serum concentration level of the ten outpatient visits (Table 2) revealed that standard deviation for mean TSH level was much higher in permanent CH cases. In fact, 54.9 % of permanent CH patients had experienced at least one uncontrolled TSH level (TSH ≥ 5) at some point during the three years of follow up vs. the 36 % in transient CH cases. Similar results were obtained by Razavi et al., in Hamadan where TSH serum concentration level during the three years of follow up were significantly higher in the permanent CH subjects. (6.10 ± 6.18 vs. 2.92 ± 3.50 mIU/L in permanent and transient CH respectively, *P* < 0.001) [19].

Association between TSH serum concentration level at screening tests and the venous sampling with transient vs. permanent CH were inconsistent. Many studies found TSH serum concentration level at screening and the first venous sampling to be significantly higher in permanent CH [17, 19, 24, 32, 35]; while only a few studies found no significant difference between the two groups.[34, 36] We found TSH serum concentration level > 43.35 mIU/L in the venous sampling (initial TSH) with the sensitivity of 31.66 % and specificity of 90.32 % to be the best cutoff point among the three TSH measurements in this study. Zdraveska et al., found an initial TSH level of < 30.5 mIU/L to be the predictor for transient CH with sensitivity of 92 % and specificity of 75.6 % [37].

Several cutoffs have been proposed for better predicting permanent or transient CH, using levothyroxine dose at the third year including cutoffs of > 2.86 µg/kg by Park ES et al., (Sensitivity: 88.9 %, Specificity: 71 %) [38] and > 3.96 µg/kg by Itonaga et al., (Specificity: 100 %) [39] for permanent CH and cutoffs of < 1.3 µg/kg by Higuchi et al., (Sensitivity: 80 %, Specificity: 84 %) [36] and < 2.76 µg/kg by Park IS et al., (Sensitivity: 87.3 %, Specificity: 67.6 %) [40] for transient CH. In our study, a cutoff point of > 2.25 µg/kg with a sensitivity of 76.11 % and specificity of 58.52 % at the third year of follow up was the predictor for permanent CH. However, it is important to note that despite statistical significance in the predictive values of our study, the low AUCs as demonstrated in Table 3 indicate low pragmatic values in the cut-offs for the prediction of permanent CH.

The main limitation of this study is that despite recalls, we did not have access to follow-up data for more than 200 CH cases. As the screening program will improve overtime, further study on patient follow up with more thorough data is warranted. Another limitation of this study is the lack of etiologic investigations for confirmed CH cases. This is due to the ‘National Congenital Hypothyroidism Screening Program Guidelines’ which do not require physicians to perform investigations such as thyroid scan or ultrasonography for CH etiologic factors. Exclusion of thyroid agenesis and ectopia could have provided a more meaningful predictive values for patients with eutopic thyroid glands.

## Conclusions

According to our study, we found that Fars province has one of the highest incidence of CH in Iran. Meanwhile, the transient CH rates in Fars and other parts of Iran appear to be much higher than the historical proportions of approx. 20 % transient disease in the rest of the world. Despite higher TSH concentrations for permanent CH at various points of follow-up, differences in the trend of TSH reduction after outpatient visits were insignificant between the permanent and transient CH cases. TSH concentration at both screening tests and the venous sample along with LT4 dose per kilogram at the end of second and third year of follow-up were the predictors for permanent CH.

## Data Availability

The datasets used during the current study are confidential and not publicly available. Data requests are only available upon permission from the university and the office of non-communicable diseases.

## Abbreviations

SUMS: Shiraz University of Medical Sciences
CH: Congenital hypothyroidism
AUC: Area under curve
LT4: Levothyroxine
TSH: Thyroid stimulating hormone
T4,: Thyroxine
